# Role of outpatient parenteral antibiotic therapy in the treatment of community acquired skin and soft tissue infections in Singapore

**DOI:** 10.1186/s12879-017-2569-4

**Published:** 2017-07-06

**Authors:** Monica Chan, Chee Kheong Ooi, Joshua Wong, Lihua Zhong, David Lye

**Affiliations:** 1 Department of Infectious Diseases, Institute of Infectious Diseases and Epidemiology, Tan Tock Seng Hospital, 11 Jalan Tan Tock Seng, Singapore, 308433 Singapore; 2 Department of Emergency Medicine, Tan Tock Seng Hospital, Singapore, Singapore; 3 Outpatient Parenteral Antibiotic Therapy Clinic, Tan Tock Seng Hospital, Singapore, Singapore; 4 Lee Kong Chian School of Medicine, Nanyang Technological University, Singapore, Singapore; 5 Yong Loo Lin School of Medicine, National University of Singapore, Singapore, Singapore

**Keywords:** Cellulitis, Outpatient, Parenteral antimicrobial therapy, Infection, Antibiotic, Treatment

## Abstract

**Background:**

Treatment of community acquired skin and soft tissue infections (SSTIs) is a common indication for outpatient parenteral antibiotic therapy (OPAT) in USA, UK and Australasia, however data from Asia are lacking. OPAT is well established within the Singapore healthcare since 2002, however, systematic use of OPAT for the treatment of SSTIs remains infrequent. In this report, we describe the treatment and outcome of patients with SSTIs referred directly from Emergency Department (ED) to OPAT for continuation of intravenous (IV) antibiotics in Singapore, thus avoiding potential hospital admission.

**Method:**

This is a single center university hospital retrospective study of patients with SSTIs presenting to ED who were assessed to require IV antibiotics and accepted to the OPAT clinic for continuation of IV treatment. Exclusion criteria were: haemodynamic instability, uncontrolled or serious underlying co-morbidities, necessity for inpatient surgical drainage, facial cellulitis and cephalosporin allergy. Patients returned daily to the hospital’s OPAT clinic for administration of IV antibiotics and review, then switched to oral antibiotics on improvement.

**Results:**

From 7 February 2012 to 31 July 2015, 120 patients with SSTIs were treated in OPAT. Median age was 56 years and 63% were male. Lower limbs were affected in 91%. Diabetes was present in 20%. Sixty-seven (56%) had been treated with oral antibiotics for a median duration of 3 days prior to OPAT treatment. Common symptoms were erythema (100%), swelling (96%), pain (88%) and fever (55%). Antibiotics administered were IV cefazolin with oral probenecid (71%) or IV ceftriaxone (29%) for median 3 days then oral cloxacillin (85%) for median 7 days. Clinical improvement occurred in 90%. Twelve patients (10%) were hospitalized for worsening cellulitis, with 4 patients requiring surgical drainage of abscess. Microbiological cultures from 2 patients with drained abscess grew *methicillin sensitive Staphylococcus aureus* (MSSA) and *Klebsiella pneumoniae*, both of which were susceptible to cefazolin and ceftriaxone. Hospital bed days saved was 318 days.

**Conclusion:**

OPAT treatment of community acquired SSTIs in a selected patient population has good outcomes in Singapore. Treatment with IV cefazolin or IV ceftriaxone was successful in the majority with low hospital readmission rate.

## Background

Outpatient parenteral antibiotic therapy (OPAT) is the provision of at least two doses of intravenous (IV) antibiotics without intervening hospital stay. Since 1974, when OPAT was first reported for the treatment of pediatric patients with cystic fibrosis [1], the practice has been adopted worldwide [2–4]. Outpatient parenteral antibiotic therapy is a safe and cost-effective means of avoiding or reducing length of hospital stay for patients who require IV antibiotics but are otherwise medically stable.

Skin and soft tissue infections (SSTIs) are common indications for OPAT treatment in North America, United Kingdom and Australasia [2–4]. A significant proportion of OPAT courses administered in these countries, for example, up to 52% in a 10 year retrospective OPAT cohort in Glasgow UK, is for the treatment of SSTIs [5].

Outpatient parenteral antibiotic therapy was first introduced to Singapore in 2002 and is now a well accepted practice within the healthcare system [6]. Despite this experience, use of OPAT for the treatment of SSTIs in Singapore remains infrequent. Based on literature regarding the success, safety and cost-effectiveness of OPAT for the treatment of SSTIs, a clinical protocol was developed enabling patients diagnosed with SSTIs in the Emergency Department (ED) of a Singapore university hospital to be referred directly to OPAT, thus avoiding potential hospital admission. In this report, we describe the characteristics, treatment and outcomes of patients treated by this management protocol since 2012.

## Methods

Tan Tock Seng Hospital (TTSH) is a 1500-bed university teaching hospital and the second largest acute general hospital in Singapore. The hospital’s OPAT service was established in 2002, providing care for approximately 350 patients per year and saving an estimated 5000 bed days per year. The majority of OPAT referrals are from the hospital’s inpatient wards. After assessment by the OPAT nurse and physician, enrolled patients receive antimicrobials by either daily bolus infusion or elastomeric infusors in the hospital’s OPAT clinic (76% of episodes) or in the patient’s home by trained carers (17%) or outreach nurses (7%) [6]. The hospital’s OPAT clinic comprises four recliner chairs and one bed for infusion therapy and three consultation rooms. Both the hospital’s OPAT clinic and outreach nurses provide OPAT services daily – during office hours on weekdays and until noon on weekends and public holidays. The service is managed by a multidisciplinary team of five full time OPAT nurses, Infectious Diseases physicians and supported by the hospital pharmacy.

Tan Tock Seng Hospital has one of the busiest emergency department in Singapore, with an average of 450 attendances a day. Skin and soft tissue infections are a common diagnosis, present in approximately 350 attendances monthly, of which 50% are admitted to inpatient wards, an additional 10% are observed in short stay wards and the remaining discharged with oral antibiotics. From 2012, patients presenting to ED with SSTIs and assessed by the ED physician to require IV antibiotics were referred to the hospital’s OPAT clinic. Skin and soft tissue infections (SSTIs) were defined as a recent onset of soft tissue erythema associated with signs of infection that included ≥1 of the following symptoms: pain, swelling, fever, warmth or lymphangitis. The inclusion and exclusion criteria are stated in Table 1. Although the diagnosis of systemic inflammatory response syndrome (SIRS) was not an exclusion criteria, haemodynamic instability as assessed by tachycardia, and low systolic blood pressure < 90 mmHg were contraindications to OPAT treatment. Patients with uncontrolled significant co-morbidities such as poorly controlled diabetes, congestive cardiac failure or immunocompromised state such as active malignancy, on immunosuppressants or immunomodulatory agents were excluded. Patients with abscesses that were adequately incised and drained in ED could be accepted to OPAT, however, patients needing more extensive debridement or inpatient surgical interventions were admitted to the hospital. No minimum duration of oral antibiotic therapy was necessary prior to consideration of OPAT therapy.

**Table 1:** Inclusion and exclusion criteria

Inclusion criteria	
Cellulitis not improving with oral antibiotics Cellulitis associated with lymphangitis	
Exclusion criteria	
Hemodynamic instability i.e. heart rate > 100 beats per minute, systolic blood pressure < 90 mmHg Cellulitis where underlying bone or joint involvement cannot be excluded Cellulitis related to recent surgery Facial or periorbital cellulitis Newly diagnosed or poorly controlled diabetes mellitus Other active or poorly controlled significant co-morbidities requiring hospitalization Immunocompromised state e.g. active malignancy, immunosuppressants Penicillin or cephalosporin allergy Unable to attend hospital OPAT clinic daily	

Clinical evaluation at baseline included patient demographics, underlying co-morbidities, potential predisposing factors, and physical examination including extent of affected area. Laboratory tests done at ED included full blood count, renal function, random glucose and C-reactive protein. Radiographic imaging of the affected limb to exclude the presence of a foreign body, gas in the soft tissue, or osteomyelitis was performed where clinically indicated.

Patients referred by ED were further evaluated by the OPAT physician and counseled by experienced OPAT nurses. Information leaflets and emergency contact numbers were provided and at least one dose of IV antibiotic was administered in ED before discharge. Daily IV antibiotics were then administered via peripheral cannula in the hospital OPAT clinic from the following day. From February 2012 to November 2012, IV ceftriaxone 2 g was administered, this was subsequently switched to IV cefazolin 2 g with oral probenecid 1 g [7] from December 2012 onwards to narrow the spectrum of antimicrobial activity. Daily temperature, pulse rate, blood pressure, patient symptoms and extent of cellulitis were monitored at each visit. After approximately three days of IV antibiotics in the OPAT clinic, patients with satisfactory response to treatment, as defined by the resolution of fever, receding erythema and reduction in swelling and pain, were switched to oral antibiotics, usually cloxacillin or clindamycin for a week [8]. The duration of intravenous or oral antibiotics could be shortened or extended by the OPAT physician depending on patient response. An outpatient review was arranged after seven days of oral antibiotics, with additional follow-up arranged until completion of oral therapy if this was extended. Concomitant tinea pedis or eczema was treated with appropriate topical antifungal cream or emollients respectively. Patients not responding to IV antibiotics, with local or systemic deterioration, were alerted to the OPAT physician and appropriate management such as drainage of abscess or hospital admission was arranged. The collection and publication of these data was approved by the institutional ethics review board.

### Statistical analysis

Descriptive statistics (median, interquartile ranges [IQR], frequency counts) were used to describe the distribution of variables in the population. Fisher’s exact test was used accordingly to evaluate differences in categorical variables, while Mann-Whitney test was used for continuous variables. All tests were two-tailed and *p*-values less than 0.05 were considered statistically significant. The statistical analysis was performed using Stata version 13 (StataCorp LP, College Station, TX, USA).

## Results

One hundred and twenty patients were treated by OPAT during the 41-month period from 7 February 2012 to 31 July 2015. The majority were male (63%) and the median age was 56 years. The median duration of symptoms was four days (IQR 3–7 days). In addition to soft tissue erythema, typical symptoms included swelling (96%), pain (88%), fever or history of fever (55%) and lymphangitis (13%). Of the 66 patients with fever or history of fever, 26 patients (39.3%) had a maximum temperature ≥ 38 °C documented in ED. The most common factor predisposing to cellulitis was an abrasion or laceration in 34 patients (28%) followed by eczema (9.1%), insect bite (6.7%) and tinea pedis (6.7%). Lower limbs were affected in 91%. Diabetes was the most common co-morbidity in 20%. Sixty-seven (56%) had been treated with oral antibiotics for a median duration of three days (3 to 5 days) prior to OPAT treatment (Table 2). Although the majority of patients were treated for cellulitis, four patients had a localized abscess which were incised and drained in ED prior to OPAT.

**Table 2:** Demographic and clinical features

Variables	Completed (*n* = 108)	Re-admitted (*n* = 12)	*p*-value
Gender, male	68 (63.0)	7 (58.3)	0.76
Age, years	55 (41–67)	60 (50–65)	0.39
Co-morbidities			
Diabetes mellitus	20 (18.5)	4 (33.3)	0.26
Peripheral vascular disease	0 (0)	1 (8.3)	0.10
Renal disease	1 (0.93)	1 (8.3)	0.19
Precipitating factors			
Eczema	11 (10.2)	0 (0)	0.60
Tinea pedis	4 (3.7)	4 (33.3)	<0.01
Trauma	32 (29.6)	2 (16.7)	0.51
Insect bite	8 (7.4)	0 (0)	1
Venous insufficiency	2 (1.9)	1 (8.3)	0.27
Prior antibiotic treatment	63 (58.3)	4 (33.3)	0.13
Prior antibiotic duration, days	3 (3–5)	2 (1–5)	0.42
Site of cellulitis			
Lower limb	97 (89.8)	12 (100)	NA
Upper limb	11 (10.2)	0 (0)	0.60
Clinical features			
Duration of symptoms, days	4 (3–7)	3 (3–7)	0.93
Swelling	104 (96.3)	11 (91.7)	0.42
Pain	93 (86.1)	12 (100)	0.36
Lymphangitis	13 (12.0)	2 (16.7)	0.65
Ulceration	7 (6.5)	1 (8.3)	0.58
Fever or reported fever	56 (51.9)	10 (83.3)	0.06
Max temperature, ^o^C	37.2 (36.9–37.7)	37.6 (37.1–38.3)	0.19

For categorical variables, data was presented in frequencies and percentages. Fisher’s exact test was used to test for association

For continuous variables, data was presented in median and interquartile range. Mann-Whitney test was used to test for differences

Baseline blood tests showed leukocytosis (10.4 × 10^9^/L [7.8–12.7]) and elevated CRP (23.3 mg/L [10.5–55.7]). Intravenous antibiotics administered were ceftriaxone in 35 patients (29%) from February 2012 to November 2012 and cefazolin in 85 patients (71%) from December 2012 to July 2015. Intravenous antibiotics were administered for a median duration of three days, defined as the days of IV antibiotics administered in the hospital’s OPAT clinic, followed by oral cloxacillin (85%) or clindamycin (4%) for a median duration of seven days (Table 3).

**Table 3:** Treatment and outcome

Variable	Completed (*n* = 108)	Re-admitted (*n* = 12)	*p*-value
Laboratory findings (initial)			
WBC, ×10^9^/L	10.4 (7.8–12.9)	11.0 (9.1–12.4)	0.66
C-reactive protein, mg/L	21.8 (8–50)	74 (17–147)	0.17
Laboratory findings (end of treatment)			
WBC, ×10^9^/L	7.7 (6.3–9.0)	9.1 (7.4–10.9)	0.21
C-reactive protein, mg/L	3.2 (1.3–6.7)	10.1 (1.9–22.5)	0.14
IV antibiotics used			
Duration of IV antibiotics, days	3 (3–3)	3 (3–3)	0.54
Cefazolin	77 (71.3)	8 (66.7)	0.74
Ceftriaxone	31 (28.7)	4 (33.3)	NA
PO antibiotics used			
Duration of PO antibiotics, days	7 (7–7)	7 (6–12)	0.26

For categorical variables, data was presented in frequencies and percentages. Fisher’s exact test was used to test for association

For continuous variables, data was presented in median and interquartile range. Mann-Whitney test was used to test for differences

Of 120 patients that were treated in OPAT, clinical improvement occurred in 90%. Twelve patients (10%) required hospital admission. Seven had worsening cellulitis requiring further course of IV antibiotics. Four patients admitted required surgical incision and drainage of abscess under general anesthetic. Intra-operative cultures were positive for methicillin sensitive *Staphylococcus aureus* (MSSA) and *Klebsiella pneumoniae* in one patient each. Both organisms were susceptible to cefazolin and ceftriaxone. One patient was admitted for acute renal impairment following administration of five days of ceftriaxone, one week of oral amoxicillin-clavulanic acid then one week of oral cloxacillin. The renal impairment was attributed to antibiotic related interstitial nephritis and resolved after cessation of antibiotics. Two patients required outpatient surgical drainage of abscess under local anesthetic in the ED but both did not require hospital admission and recovered after an additional course of oral antibiotics. The total number of hospital beds days saved, calculated as the number of days for which IV antibiotics was administered in the hospital’s OPAT clinic, was 318 days in total, with median of 6 days (4–8 days) saved per month.

Patients requiring hospital re-admission were more likely to have a diagnosis of tinea pedis compared to those who completed treatment (33.3% vs. 3.7%, *p* < 0.01), however, these patients had a diagnosis of concomitant trauma or diabetes which may have additionally contributed to the readmission. A trend towards higher median CRP at baseline was noted in patients readmitted compared to those who completed treatment (74 vs. 21.8, *p* = 0.17), but did not reach statistical significance. Other characteristics including age, co-morbidities, duration of symptoms were not significantly different between the two groups.

Patients treated with antibiotics prior to ED attendance tended to have sustained more trauma (35.8% vs 18.9%, *p* < 0.01), more ulceration (11.9% vs 0%, *p* < 0.01), and had longer duration of symptoms (median 6 days vs 3 days) compared to patients without prior antibiotic exposure (Table 4). Otherwise baseline characteristics, treatment and outcome were similar. There was no significant difference in baseline characteristics in patients treated with IV ceftriaxone 2 g once daily compared to IV cefazolin 2 g once daily with oral probenecid 1 g. Median duration of IV therapy, subsequent oral antibiotic treatment, and successful completion of treatment (90.5% vs. 85.7%, *p* = 0.5) were also similar for both groups.

**Table 4:** Comparison of patients treated with antibiotics prior to Emergency Department attendance

Variables	Prior antibiotics (*n* = 67)	No prior antibiotics (*n* = 53)	*p*-value
Trauma	24 (35.8)	10 (18.9)	0.04
Ulceration	8 (11.9)	0 (0)	<0.01
Duration of symptoms, days	6 (4–7)	3 (2–4)	<0.01
Max temperature, ^o^C	37.1 (36.8–37.3)	37.4 (37.1–38.3)	<0.01

For categorical variables, data was presented in frequencies and percentages. Fisher’s exact test was used to test for association

For continuous variables, data was presented in median and interquartile range. Mann-Whitney test was used to test for differences

## Discussion

In this report, we describe the successful outcomes of OPAT treatment in patients with SSTIs referred directly from ED to OPAT for continuation of IV antibiotics, without intervening hospital admission in a Singapore university hospital. Although a common source of OPAT referrals in US, UK and Australasia, there is a lack of published data from Asia regarding the use of OPAT in treating SSTIs.

Prior to the implementation of the referral workflow, approximately 9 patients with SSTIs were treated each year in our OPAT clinic. In contrast, 120 patients were treated in the 3 years since implementation, saving 318 hospital bed days. In our study, overall success was 90% with hospital admission in 10%. These outcomes compare favorably with published literature. Of 963 patients with cellulitis treated in Glasgow, UK, success rate was 87%, hospital admission occurred in 6%, adverse events in 7% with progression of infection in 2.8% [9]. In Alberta, Canada, a five-centre ED retrospective study of 416 patients, 11% required change in antibiotic therapy and 7% were hospitalized [10]. A randomized controlled trial in Christchurch, New Zealand comparing cellulitis treatment at home or in the hospital had 11.8% readmission rate for 101 patients on home treatment [11].

There are no standard criteria for when IV antibiotics are necessitated versus oral antibiotics for cellulitis, with studies having differing thresholds of severity. However, 56% of our patients had previously failed oral antibiotics prior to ED attendance, comparable to 51% to 71% of patients reported in other studies [11, 12] and indicates our threshold for giving IV antibiotics was appropriate.

Similarly, no validated objective measures exist of when patients can be switched from parenteral to oral antibiotics. Practice guidelines from the USA advocate once or twice weekly patient review by physician whilst undergoing OPAT but do not specify frequency of review for patients with cellulitis [13]. More recent UK guidelines suggest daily review in OPAT treatment of SSTIs to allow rapid switch from IV to oral therapy as soon as clinically indicated [2]. In UK, IV antibiotics were administered in OPAT for a median duration of 3 days in Glasgow, mean of 5.3 days in Dundee and 6.3 days in Sheffield [9, 14, 15]. Elsewhere, duration of IV antibiotics ranged from 6.24 days to 7 days in Australia [12, 16] to median 4 days in Canadian emergency rooms [10]. The median duration of antibiotics in our study was 3 days, which compares favorably with these studies and consistent with expert recommendations suggesting patients with uncomplicated infections should be switched after 3 to 4 days of IV therapy [8].

Previous studies have investigated factors associated with OPAT failure. In a retrospective cohort, female sex, diabetes and treatment with teicoplanin were independently associated with OPAT failure [9]. Longer duration of IV therapy was associated with methicillin-resistant *Staphylococcus aureus* (MRSA), older age, vascular disease, diagnosis of bursitis, treatment with teicoplanin in one cohort [9] and male sex, high baseline CRP and prolonged duration of symptoms prior to OPAT in another cohort [15]. Due to the small numbers in this report, we did not detect any significant difference in these risk factors for our cohort.

Most cases of community-acquired cellulitis are caused by *Staphylococcus aureus* and β-hemolytic streptococci. However, microbiologic isolation of pathogen is usually not possible in the majority. Consequently, cellulitis is generally treated empirically, with therapy usually directed towards these common pathogens unless otherwise indicated [17]. Of the 2 patients with positive cultures obtained from drained abscesses, MSSA and *Klebsiella pneumoniae* were isolated, confirming that cefazolin or ceftriaxone was appropriate in our management protocol for empirical treatment of community-acquired SSTI in Singapore. No cases of MRSA was isolated in keeping with the low prevalence of community acquired MRSA in Singapore [18].

Although our study was not designed to compare outcomes between ceftriaxone and cefazolin treated patients, no significant differences in outcome were noted between the two groups. A randomized double-blind controlled trial of once daily cefazolin 2 g with probenecid 1 g compared to once daily ceftriaxone 1 g for moderate to severe cellulitis found no difference in clinical cure at end of treatment (86% vs. 96%, *p* = 0.11) and at 1 month follow-up (96% vs. 91%, *p* = 0.55) [7]. In a retrospective study of 122 patients with documented MSSA infections treated in OPAT, 64% patient had cefazolin and 36% received ceftriaxone in OPAT. Favorable clinical outcomes were similar between cefazolin and ceftriaxone (67.9% vs. 79.8%, *p* = 0.17) with a similar incidence of adverse events and complications (5.1% vs. 2.3%, *p* = 0.65 and 26.9 vs. 18.2% *p* = 0.38) [19].

Our study has several limitations. Firstly, the lack of a standard severity scale for assessment of SSTIs may lead to a potential variance in the need for IV antibiotics and duration of antibiotics required. Reassuringly, the data from our study is broadly similar to findings of other outpatient cohorts of SSTIs treated with IV antibiotics. Secondly, as patients were managed as part of a clinical protocol, follow-up blood tests and microbiological investigations were not mandated for all patients resulting in incomplete data particularly on follow-up. Treatment related side effects were also not systematically recorded. However, aside from one patient with antibiotic related interstitial nephritis, none of the remaining patients developed significant adverse reactions necessitating a change in antibiotic therapy.

## Conclusion

In conclusion, our report supports that OPAT treatment of community acquired SSTIs in a selected patient population has good outcomes in Singapore. Treatment was successful in the majority with low readmission rates comparable to other studies internationally.
